# Early and Late Prognostic Factors in Patients with Resected Brain Metastases

**DOI:** 10.3390/cancers18193157

**Published:** 2026-09-29

**Authors:** Dan M. Visarion, Ioana Petrescu, Viorel M. Pruna, Radu M. Gorgan

**Affiliations:** 1 Department of Neurosurgery, “Carol Davila” University of Medicine and Pharmacy, 020021 Bucharest, Romania; marius-dan.visarion@drd.umfcd.ro (D.M.V.);; 2 Department of Neurosurgery, “Bagdasar-Arseni” Clinical Emergency Hospital, 041915 Bucharest, Romania; mironioana@gmail.com

**Keywords:** postoperative survival predictors, brain metastases, prognostic factors, 30-day mortality, secondary brain tumor

## Abstract

Background: Brain metastases are the most common malignant intracranial tumors and remain associated with poor survival despite advances in multimodal treatment. This study aimed to evaluate the associations between clinical, radiological, surgical, and perioperative characteristics and outcomes in patients undergoing surgical resection of brain metastases. Materials and Methods: This retrospective single-center cohort study included 378 consecutive adult patients who underwent resection of histopathologically confirmed brain metastases between 2015 and 2023. Overall survival was estimated using the Kaplan–Meier method, and differences between groups were assessed using the log-rank test. Factors associated with overall survival were evaluated using univariable and multivariable Cox proportional hazards regression. Binary logistic regression was used to evaluate factors associated with 30-day mortality. Results: Median overall survival was 11.3 months, the one-year survival rate was 45.5%, and 18 patients (4.8%) died within 30 days after surgery. In the adjusted logistic regression model, preoperative KPS < 80, hydrocephalus, postoperative hematoma, and postoperative pneumonia were associated with higher odds of 30-day mortality. In the multivariable Cox regression analysis, increasing age, preoperative KPS < 80, tumor volume ≥ 9 cm^3^, hydrocephalus, wound complications, and postoperative KPS deterioration were independently associated with shorter overall survival. Subtotal versus total resection was not significantly associated with overall survival after adjustment. Conclusions: Several preoperative and postoperative characteristics were associated with early mortality and overall survival in this surgically selected cohort. These findings emphasize the importance of careful patient selection, preservation of functional status, optimization of perioperative care, and prevention of postoperative complications.

## Simple Summary

Brain metastases are among the most common intracranial tumors in adults, and outcomes after surgical treatment vary considerably among patients. This retrospective study analyzed 378 consecutive adults who underwent resection of histologically confirmed brain metastases at Bagdasar-Arseni Clinical Emergency Hospital between 2015 and 2023. Median overall survival was 11.3 months, and 18 patients died within 30 days after surgery. In multivariable analysis, poor preoperative functional status, hydrocephalus, postoperative hematoma, and postoperative pneumonia were associated with higher 30-day mortality. Increasing age, poor preoperative functional status, tumor volume ≥ 9 cm^3^, hydrocephalus, postoperative wound complications, and worsening functional status during the first postoperative week were independently associated with shorter overall survival. However, the tumor-volume finding should be interpreted cautiously because it depended on the selected cutoff. These findings highlight the importance of careful patient selection, preservation of functional status, optimization of perioperative care, and early prevention and management of postoperative complications. As this study included only surgically treated patients, its findings represent prognostic associations within this population and do not establish a survival benefit attributable to surgery itself.

## 1. Introduction

The landscape of neuro-oncology is increasingly challenged by brain metastases, which represent the most frequent intracranial malignancies in adults [1,2]. This rising incidence is likely attributable to population aging and improved systemic treatments that prolong patient survival, thereby increasing the likelihood of central nervous system involvement [1]. Throughout the course of the disease, approximately 20–30% of patients with cancer are diagnosed with brain metastases [3,4], although this proportion is likely underestimated. Recent studies indicate that up to 15% of patients are diagnosed with brain metastases without concurrent identification of the primary malignancy. In a proportion of these cases, systemic cancer cannot be localized; these individuals are classified as patients with brain metastases from cancer of unknown primary (CUP) [5]. The primary malignancies most frequently associated with brain metastases include lung cancer, affecting 20–56% of these patients, followed by breast cancer (5–20%) and melanoma (7–16%) [2,6]. Notably, a significant proportion of these individuals (40–60%) present with multiple intracranial lesions at the time of the initial diagnosis [1,3]. Overall survival (OS) rates for patients with brain metastases remain low, with only 5–24% of patients surviving up to two years and only 2.4–15% reaching the five-year mark, regardless of the primary tumor type [7,8].

Multiple prognostic factors are documented in the literature, including sex, clinical status on admission, age, tumor origin, gross total resection, molecular subtype, and therapeutic modalities, which collectively aid in patient stratification [3]. A deeper understanding of both these well-documented variables and newly identified factors influencing the incidence and progression of brain metastases holds the potential to substantially reshape clinical management and elevate therapeutic efficacy [9].

## 2. Materials and Methods

**Figure 1:**
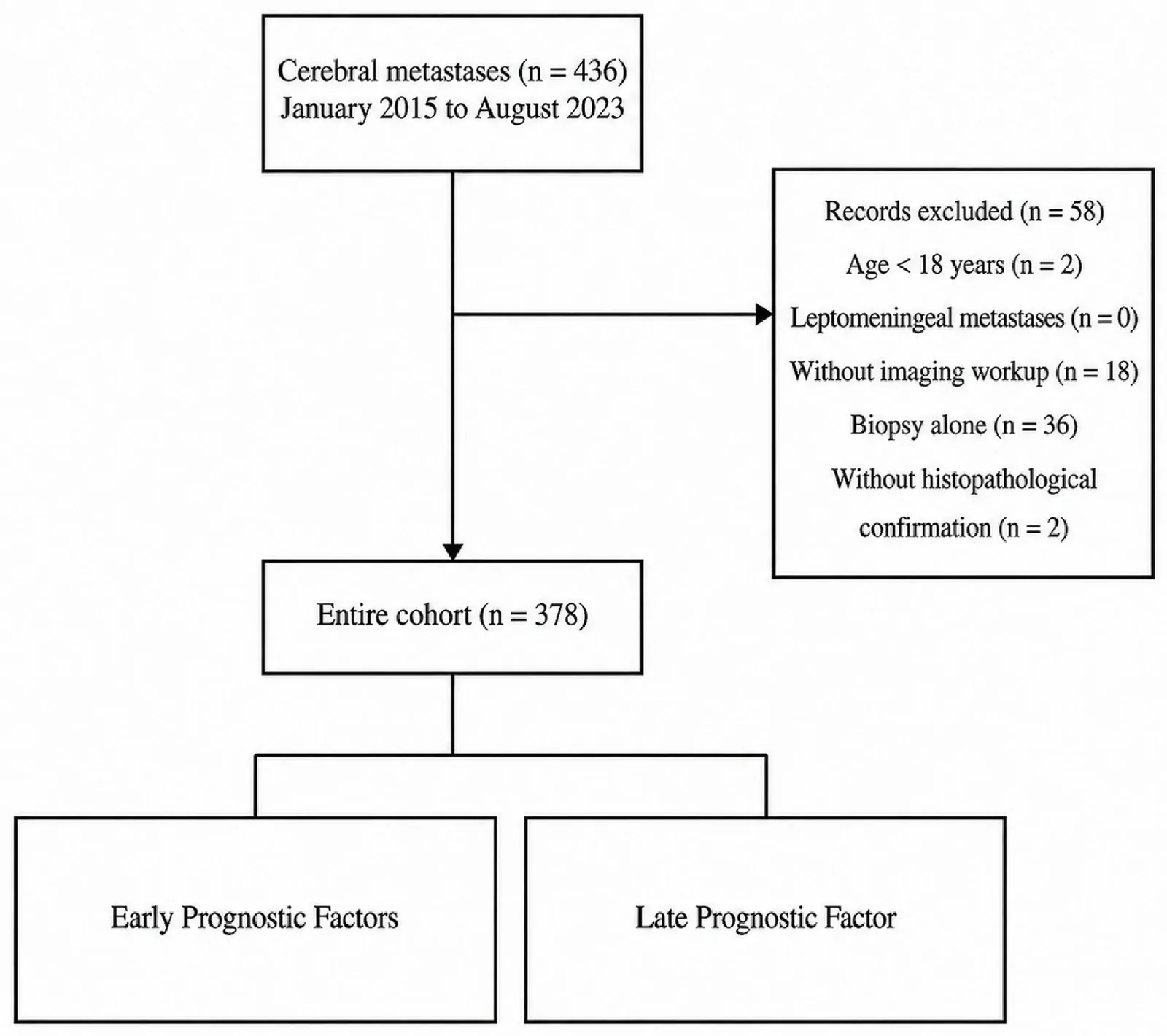
Flowchart illustrating the design of the study.

This retrospective single-center cohort study included 436 consecutive adult patients operated for brain metastases at the Bagdasar-Arseni Emergency Clinical Hospital between January 2015 and August 2023. After applying the exclusion criteria, the final cohort encompassed 378 patients with histologically confirmed brain metastases (Figure 1). Patients with leptomeningeal metastases were considered ineligible, although no such cases were identified in our database.

Demographic, clinical, imaging, surgical, and postoperative data were retrospectively collected from institutional medical records. The evaluated variables included age, sex, primary tumor type, neurological deficits at presentation, comorbidities, preoperative and postoperative Karnofsky Performance Status (KPS), number and location of brain metastases, tumor volume, extent and type of resection, and postoperative complications.

Neurological deficits were categorized as motor, sensory, speech, or coordination disturbances. Metastatic burden was categorized according to the number of intracranial lesions (1, 2–3, or ≥4) and their anatomical distribution (supratentorial, infratentorial, or both).

Preoperative KPS was assessed at admission and categorized as <80 or ≥80. Postoperative KPS was reassessed on postoperative day 7. Postoperative KPS deterioration was defined as a decrease of at least 10 points from the admission value. Patients who died before the scheduled postoperative assessment were assigned a KPS score of 0 and classified as having postoperative KPS deterioration.

Metastases were also classified as synchronous and metachronous. Overall survival was calculated from the date of surgery to death from any cause or the last available follow-up. Thirty-day mortality was defined as death from any cause occurring within 30 days of surgery.

Preoperative 1.5-T MRI examinations were retrospectively reviewed and included precontrast and postcontrast T1-weighted imaging, T2-weighted imaging, T2-FLAIR, diffusion-weighted imaging, and ADC maps. The evaluated imaging characteristics included lesion number and location, enhancement pattern, peritumoral edema, intratumoral hemorrhage, and the presence of necrotic or cystic components.

MRI examinations were evaluated by a specialist radiologist and a neurosurgeon. The volume of the surgically treated index metastasis was estimated manually on thin-section, three-dimensional, contrast-enhanced T1-weighted images using the ellipsoid formula: V = A × B × C × π/6, where A, B, and C represented the maximum orthogonal tumor diameters. Necrotic or cystic components enclosed within the contrast-enhancing tumor margin were included in the calculation. In patients with multiple brain metastases, the volume of the surgically treated index lesion was analyzed rather than the cumulative intracranial tumor volume.

The 9 cm^3^ cutoff represented the whole-number value closest to the median volume of the index lesions and was used to obtain two approximately balanced groups: <9, and ≥9 cm^3^.

Surgical variables included the operative approach, en bloc versus piecemeal resection, and extent of resection, categorized as total or subtotal. All patients underwent noncontrast cerebral CT within 24 h after surgery to identify acute complications, including postoperative hemorrhage, edema, and hydrocephalus. Early postoperative contrast-enhanced MRI was available for only a limited number of patients and was not performed according to a standardized protocol. Consequently, the extent of resection was determined primarily based on intraoperative macroscopic assessment and the operative report, together with the available postoperative imaging.

Available information regarding previous and postoperative oncological treatments was reviewed. However, many patients subsequently received oncological care at other institutions, and standardized data regarding postoperative radiotherapy, systemic treatment, treatment timing, and local intracranial control were not consistently available.

Statistical analyses were performed using Statisticalanalyses were performed using IBM SPSS Statistics for Windows, version 30.0 (IBM Corp., Armonk, NY, USA). Continuous variables were summarized using the median and interquartile range, while categorical variables were reported as frequencies and percentages. All statistical tests were two-sided, and a *p*-value < 0.05 was considered statistically significant.

Overall survival was calculated from the date of surgery to the date of death or last follow-up. Survival was evaluated using the Kaplan–Meier analysis, and differences between groups were assessed using the log-rank test. Univariable and multivariable Cox proportional hazards regression analyses were performed to identify factors associated with overall survival.

The proportional hazards assumption was assessed graphically for categorical covariates included in the Cox model. Kaplan–Meier survival curves were visually inspected for substantial crossing or marked changes in their separation over time.

Factors associated with 30-day mortality were evaluated using binary logistic regression. Model calibration and discriminatory ability were assessed using the Hosmer–Lemeshow test and the area under the ROC curve, respectively. Owing to the limited number of events, this analysis was considered exploratory.

Kaplan–Meier survival curves were realized by using GraphPad Prism version 10.0 (GraphPad Software, Boston, MA, USA).

## 3. Results

The study included 378 patients who met the eligibility criteria. The cohort had a median age of 60 years (range, 18–83 years). The study cohort comprised 157 females (41.5%) and 221 males (58.5%) who underwent surgical resection for brain metastases. The baseline clinical and demographic characteristics of the entire cohort are detailed in Table 1.

### 3.1. Perioperative Factors Associated with 30-Day Mortality

Univariable binary logistic regression analyses were performed to evaluate the associations between demographic characteristics, preoperative clinical factors, postoperative complications, and 30-day mortality. Male sex (OR = 6.049, 95% CI 1.370–26.697; *p* = 0.018) and hydrocephalus (OR = 5.128, 95% CI 1.539–17.083; *p* = 0.008) were associated with higher odds of mortality, whereas KPS ≥80 showed a protective association (OR = 0.104, 95% CI 0.029–0.365; *p* < 0.001). Age, analyzed as a continuous variable, was not significantly associated with mortality (OR per one-year increase = 1.028, 95% CI 0.973–1.086; *p* = 0.321). Postoperative hematoma (OR = 22.273, 95% CI 7.144–69.444; *p* < 0.001) and postoperative pneumonia (OR = 34.231, 95% CI 8.220–142.542; *p* < 0.001) showed the strongest unadjusted associations, although these represent postoperative factors associated with early mortality rather than baseline prognostic factors.

Given the limited number of deaths within 30 days (*n* = 18), the multivariable model was restricted to four variables considered clinically relevant to a surgical cohort: KPS on admission, hydrocephalus, postoperative hematoma, and postoperative pneumonia. After simultaneous adjustment, KPS ≥80 remained protectively associated with mortality, whereas hydrocephalus, postoperative hematoma and postoperative pneumonia remained associated with higher odds of mortality, as shown in Table 2 The model was statistically significant (χ^2^ = 59.514; *p* < 0.001), showed no evidence of lack of fit according to the Hosmer–Lemeshow test (*p* = 0.469), and demonstrated excellent apparent discrimination (AUC = 0.925, 95% CI 0.875–0.976). Given the limited number of events and the wide confidence intervals, these findings should be considered exploratory and interpreted with caution.

### 3.2. Perioperative Factors Associated with Overall Survival

#### 3.2.1. Kaplan–Meier Analysis

The median overall survival (OS) for the entire cohort was 11.3 months, with a one-year survival rate of 45.5% (172 patients). Eighteen patients died within 30 days after surgical resection.

**Figure 2:**
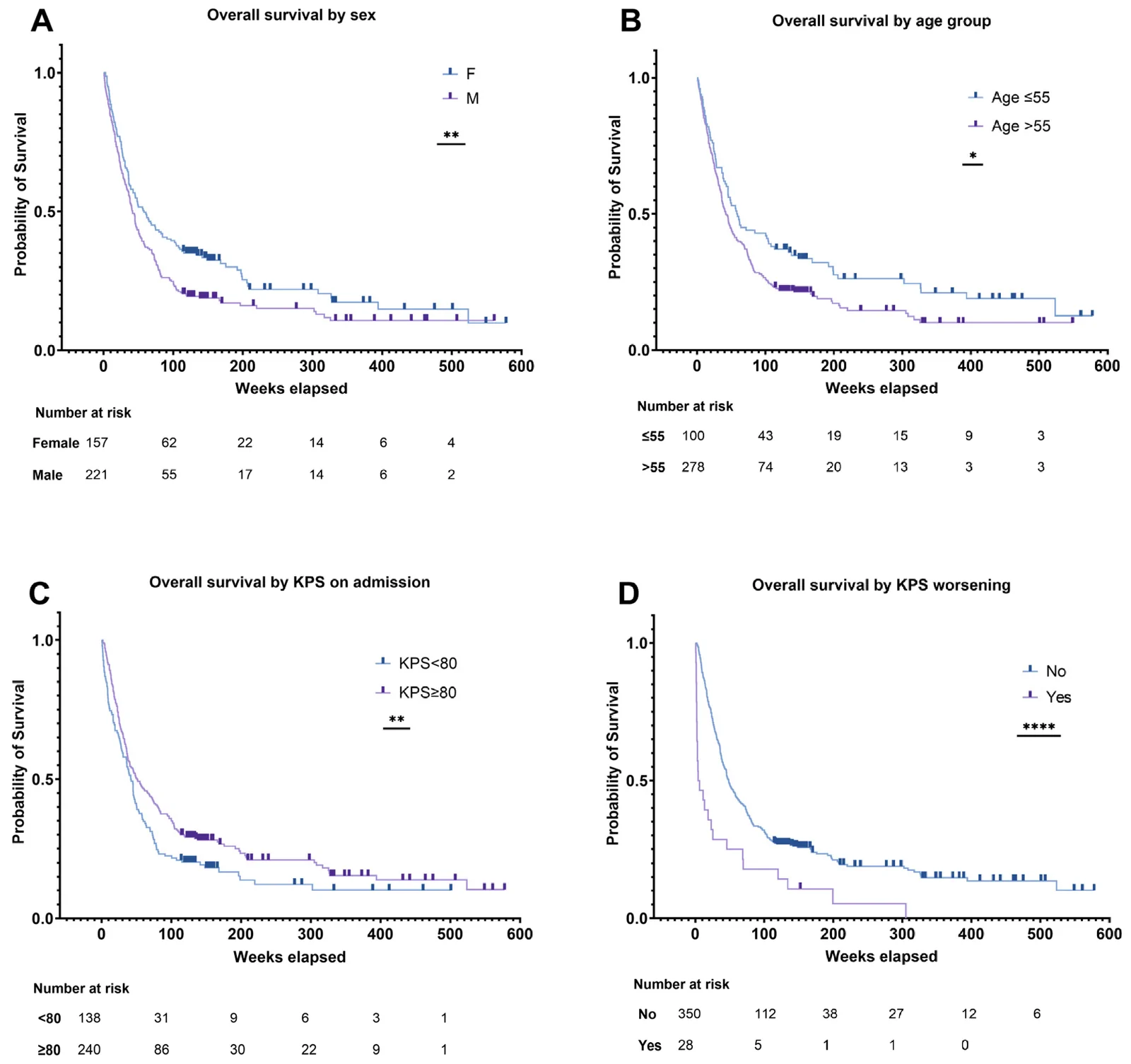
Kaplan–Meier analysis of overall survival according to different factors. (**A**) Female sex positively influenced overall survival. (**B**) Patients younger than 55 had a better overall survival compared to the older ones. (**C**) KPS ≥ 80 on admission was a favorable prognostic factor. (**D**) Survival curves according to KPS worsening after surgery. Statistical significance was indicated using the following symbols: ns for *p* > 0.05, * for *p* ≤ 0.05, ** for *p* ≤ 0.01, *** for *p* ≤ 0.001, and **** for *p* ≤ 0.0001.

Univariable Kaplan–Meier analyses demonstrated significant differences in overall survival according to several demographic and clinical characteristics. Female patients had significantly longer overall survival than male patients (*p* = 0.007), while patients aged ≤55 years showed superior survival compared with those aged >55 years (*p* = 0.026). Baseline functional status was also associated with outcome, with patients presenting a KPS ≥ 80 demonstrating significantly longer survival than those with a KPS < 80 (*p* = 0.012). Patients with postoperative KPS worsening had significantly shorter overall survival than patients without postoperative functional deterioration (*p* < 0.001) (Figure 2).

**Figure 3:**
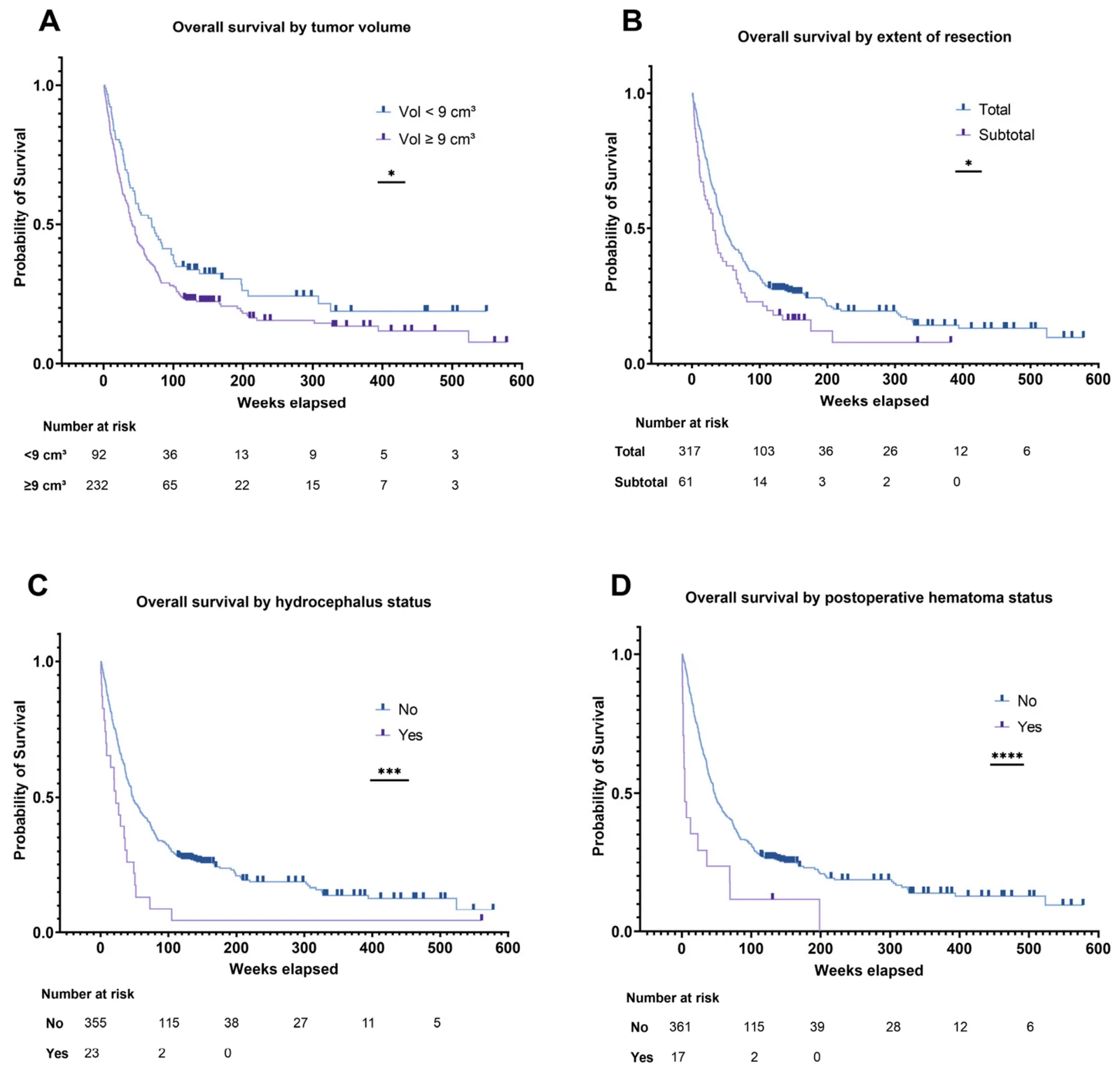
Kaplan–Meier analysis of overall survival according to different factors. (**A**). Tumor volume ≥ 9 cm^3^ was associated with significantly shorter overall survival. (**B**) Cases where total resection was possible had a significantly better overall survival. (**C**) Survival curves according to the presence of hydrocephalus on admission. (**D**) Postoperative hematoma was a negative prognostic factor. Statistical significance was indicated using the following symbols: ns for *p* > 0.05, * for *p* ≤ 0.05, ** for *p* ≤ 0.01, *** for *p* ≤ 0.001, and **** for *p* ≤ 0.0001.

Significant differences in survival were also observed according to tumor and treatment-related characteristics. The extent of surgical resection was significantly associated with OS, with patients undergoing gross total resection demonstrating longer survival than those undergoing subtotal resection (*p* = 0.017). A tumor volume ≥ 9 cm^3^ was associated with significantly reduced mean and median survival compared with a tumor volume < 9 cm^3^ (*p* = 0.023). Postoperative CSF fistula was associated with an over 80% reduction in mean survival (*p* = 0.005), while postoperative hematoma was associated with an almost fourfold reduction in mean survival and a markedly shorter median OS (*p* < 0.001). Patients who present with hydrocephalus had a median OS of 5.5 months, compared with 11.9 months among those without hydrocephalus (*p* < 0.001) (Figure 3).

The MRI contrast-enhancement pattern was also associated with survival. A significant difference was identified between lesions with punctate and homogeneous enhancement (*p* = 0.041), whereas the remaining pairwise comparisons were not statistically significant. Among the comorbidities evaluated, atrial fibrillation/flutter was associated with shorter survival, although the difference was only borderline significant (*p* = 0.057). Obesity was associated with significantly reduced mean and median survival compared with the absence of obesity (*p* = 0.013).

Several additional factors that could potentially influence overall survival were evaluated using Kaplan–Meier analysis. No statistically significant differences in survival were observed according to the timing of brain metastasis development (synchronous vs. metachronous; *p* = 0.815), the presence of extracranial metastases (*p* = 0.315), or the number of brain metastases (solitary vs. multiple; *p* = 0.558). Although patients without extracranial metastases had a numerically longer median overall survival than those with extracranial metastases 49.29 [95% CI: 39.33–59.24] vs. 41.71 [95% CI: 33.06–50.37], respectively, this difference did not reach statistical significance.

Univariable Cox proportional hazards regression analyses were performed to evaluate factors associated with overall survival, as presented in Table 3. Male sex, increasing age, preoperative KPS < 80, tumor volume ≥ 9 cm^3^, hydrocephalus, subtotal tumor resection, postoperative hematoma, postoperative CSF fistula, and postoperative KPS worsening were significantly associated with a higher risk of death. Speech impairment showed an inverse association with the risk of death (HR = 0.730, 95% CI 0.540–0.990; *p* = 0.039); however, given the counterintuitive direction and marginal statistical significance of this finding, it was considered exploratory and potentially incidental rather than evidence of a protective effect. The number of metastases and BM-CUP histology were not significantly associated with overall survival. The univariable findings were considered alongside the clinical relevance of each variable when constructing the subsequent multivariable model.

In the univariable Cox analyses, the number of brain metastases, timing of metastatic presentation, extracranial metastases, histopathological subtype, motor deficits, resection technique, and lesion location were not significantly associated with overall survival.

#### 3.2.2. Multivariable Cox Regression Analysis

To provide an integrated assessment of factors associated with overall survival, a perioperative multivariable Cox proportional hazards model was constructed using variables considered clinically relevant to this surgical cohort, including preoperative characteristics, factors present at the time of surgery, intraoperative factors, and clinical evolution during the first seven postoperative days. For the categorical covariates included in the model, the proportional hazards assumption was assessed graphically by visual inspection of the Kaplan–Meier survival curves for substantial crossing or marked changes in their separation over time. No major departures from proportionality were identified. This assessment was graphical rather than based on a formal statistical test.

The overall model was statistically significant (χ^2^ = 65.224; 7 degrees of freedom; *p* < 0.001). KPS deterioration assessed on postoperative day 7 was independently associated with a higher risk of death in the perioperative multivariable model. Hydrocephalus, wound complications, preoperative KPS < 80, tumor volume ≥ 9 cm^3^, and increasing age per additional year remained independently associated with a higher risk of death. Subtotal resection was not significantly associated with overall survival. These findings highlight the prognostic relevance of perioperative clinical status, tumor characteristics, and particularly functional evolution during the first postoperative week (Table 4).

## 4. Discussion

In this retrospective cohort of patients undergoing surgical treatment for brain metastases, we analyzed clinical, tumor-related, and therapeutic variables to determine their influence on survival outcomes. The observed median overall survival of approximately 11 months was consistent with findings from recent studies, reflecting the continued prognostic relevance of surgical intervention in this patient population. Untreated brain metastases carry a median survival of merely 1–2 months, while whole-brain radiation therapy (WBRT) as a standalone modality extends survival to approximately 4–6 months [3,5,6]. Surgical resection is an important component of multimodal management for selected patients with brain metastases, and postoperative radiotherapy can improve local control at the resection site [5,10].

### 4.1. Prognostic Factors Associated with 30-Day Mortality in the Perioperative Period

The 30-day mortality rate in our cohort was 4.8%. Male sex was associated with higher odds of mortality in the univariable analysis, whereas age, analyzed as a continuous variable, showed no significant association. After simultaneous adjustment, a preoperative KPS < 80 remained associated with significantly higher odds of 30-day mortality compared with a KPS ≥ 80. These findings are consistent with previous studies highlighting the importance of functional status in perioperative risk assessment. In this context, several studies have proposed frailty scores that integrate biological parameters, neurological status, and comorbidity burden to facilitate the early identification of patients at increased risk of mortality [11,12,13].

In addition to impaired functional status, preoperative hydrocephalus and two major postoperative complications—postoperative hematoma and postoperative pneumonia—were associated with early mortality in our cohort. After simultaneous adjustment, all three variables remained associated with significantly higher odds of 30-day mortality. These findings highlight the potential impact of both preoperative clinical status and postoperative complications on the early clinical course and underscore the importance of appropriate preoperative assessment, rigorous perioperative management, close postoperative monitoring, and prompt therapeutic intervention [7,11]. Nevertheless, given the limited number of deaths, the multivariable findings should be interpreted with caution.

### 4.2. Long-Term Prognostic Factors Following Surgical Resection of Brain Metastases

Long-term survival in patients with brain metastases is influenced by a complex interplay of factors related to the patient’s biological status, the characteristics of the underlying malignancy, and the available therapeutic options. Understanding these prognostic factors is essential for identifying patients who are most likely to benefit from radical treatment strategies and for optimizing the clinical decision-making process.

To identify factors with prognostic value for survival, multiple clinical, radiological, and treatment-related characteristics of the study cohort were evaluated. Initially, the impact of each variable on survival was assessed using KM survival analysis and comparison of survival curves. Subsequently, variables that demonstrated statistical significance in the univariable analysis, as well as those considered clinically relevant, were included in a multivariable Cox proportional hazards regression model.

This approach enabled the identification of factors independently associated with overall survival within this surgically treated cohort, after adjustment for the other covariates included in the model.

Our multivariable Cox regression analysis identified several factors independently associated with overall survival, including increasing age, preoperative KPS < 80, tumor volume ≥ 9 cm^3^, preoperative hydrocephalus, postoperative KPS worsening assessed on day 7, and postoperative wound complications. The latter represented a composite variable comprising postoperative hematoma and/or CSF leakage. Subtotal versus total tumor resection was also included in the model but did not demonstrate a significant independent association with overall survival.

#### 4.2.1. Age as an Independent Predictor of Long-Term Survival

Age has been extensively investigated as a prognostic determinant in patients with brain metastases and has been incorporated into several prognostic models developed to estimate survival in this population. In our multivariable Cox regression analysis, age was included as a continuous variable and retained its independent prognostic value after adjustment for all covariates included in the model (HR per additional year = 1.020, 95% CI 1.007–1.034; *p* = 0.003). Thus, each additional year of age was associated with an approximately 2% increase in the hazard of death. Although our findings support age as an independent prognostic factor, the available literature remains inconsistent, as several studies have failed to identify age as an independent predictor of survival after adjustment for other clinical and oncological variables [14]. These discrepancies may be related to differences in patient selection, age distribution, tumor histology, and treatment strategies across cohorts. Therefore, the prognostic effect of age should be interpreted cautiously and in conjunction with other established prognostic factors.

#### 4.2.2. The Impact of Functional Status on Survival Outcomes: The Role of Karnofsky Performance Status

In the multivariable Cox regression analysis, a preoperative KPS < 80 was independently associated with poorer overall survival compared with a KPS ≥ 80 (adjusted HR = 1.645, 95% CI 1.262–2.144; *p* < 0.001). This finding is consistent with previous studies identifying baseline functional status as an important prognostic determinant in patients with brain metastases [15,16,17].

We also evaluated early changes in functional status between the preoperative assessment and postoperative day 7. In the multivariable Cox regression model, postoperative KPS worsening was independently associated with an approximately threefold higher hazard of death compared with stable or improved functional status (adjusted HR = 3.095, 95% CI 1.892–5.063; *p* < 0.001). This finding suggests that perioperative functional decline is an important prognostic marker, potentially reflecting the combined effects of surgical morbidity, neurological deficits, postoperative complications, systemic deterioration, and reduced functional reserve, all of which may compromise recovery and subsequent oncological treatment [18].

#### 4.2.3. Prognostic Relevance and Standardization of MRI-Derived Features

In our cohort, preoperative MRI was performed using a 1.5-T system in accordance with the minimum standardized brain metastasis imaging protocol proposed by Kaufmann et al. [19]. The exploratory imaging assessment included tumor volume, number and anatomical location of metastases, contrast-enhancement pattern, perilesional edema, intratumoral hemorrhage, and intratumoral necrosis [20,21].

Among the imaging characteristics evaluated, the preoperative volume of the surgically treated index metastasis was associated with overall survival when analyzed categorically using the original 9 cm^3^ cutoff. This threshold represented the rounded median index-lesion volume in the present cohort and was not selected by optimizing the separation of the survival curves. Accordingly, it should be considered a data-dependent exploratory threshold rather than a prespecified clinical cutoff.

This value is close to the 10 cm^3^ threshold previously investigated in patients with brain metastases. Routman et al. reported that a cumulative intracranial tumor volume >10 cm^3^ provided more relevant prognostic information for survival than the number of brain metastases [22]. However, their study included a radiosurgical population and evaluated cumulative intracranial tumor volume rather than the volume of the surgically treated index lesion. Therefore, their findings provide contextual support but do not directly validate the 9 cm^3^ threshold identified in our surgical cohort. In a study more directly applicable to our population, Yang et al. demonstrated that increasing preoperative volume of the resected metastasis, analyzed as a continuous variable, was independently associated with poorer overall and progression-free survival [23].

In our cohort, however, tumor volume was not significantly associated with overall survival when modeled as a continuous variable (HR per 1 cm^3^ increase = 1.002, 95% CI 0.995–1.008; *p* = 0.620). Furthermore, the sensitivity analysis using an alternative 10 cm^3^ cutoff showed a 28.6% higher estimated hazard of death among patients with larger lesions, although this association did not reach statistical significance (HR = 1.286, 95% CI 0.987–1.675; *p* = 0.062). The preservation of the direction of the association, together with the loss of statistical significance in the continuous-volume model and when using the alternative cutoff, indicates that the categorical finding is sensitive to the selected threshold. Therefore, our results do not establish 9 cm^3^ as a universally applicable clinical or surgical cutoff. The association should be considered exploratory and interpreted in the context of a surgically selected population. Nevertheless, volumetric assessment may provide complementary prognostic information beyond conventional linear measurements and warrants further investigation in independent surgical cohorts [5,7,24].

Most authors use the maximum lesion diameter as the principal criterion, suggesting that brain metastases measuring ≤3 cm and associated with limited peritumoral edema are ideal candidates for stereotactic radiosurgery (SRS) [18]. Conversely, lesions larger than 3 cm or those associated with significant mass effect and extensive peritumoral edema appear to benefit more from surgical management [5,6].

Accordingly, smaller, well-demarcated lesions may be more amenable to en-bloc resection, which has been associated with improved local control and a lower risk of leptomeningeal dissemination [25,26,27]. Resection technique (en-bloc versus piecemeal) was not significantly associated with overall survival in our cohort.

Most of the other conventional MRI characteristics evaluated in our cohort were not significantly associated with overall survival. The absence of statistically significant associations does not exclude the biological relevance or potential prognostic value of these MRI characteristics. Surgical selection may also reduce imaging variability by preferentially including symptomatic, accessible, and resectable lesions, thereby diminishing the apparent prognostic contribution of individual MRI characteristics.

Previous studies have similarly reported heterogeneous findings regarding MRI-derived prognostic markers. Spanberger et al. identified peritumoral edema as an independent prognostic parameter in patients undergoing surgery for solitary brain metastases [28], whereas Kerschbaumer et al. found no significant association between tumor or edema volume and overall survival [29]. Furthermore, Gutsche et al. demonstrated that visually classified contrast-enhancement patterns had limited predictive performance compared with quantitative radiomic features [30]. These findings emphasize the need for standardized acquisition and assessment methods, predefined volumetric segmentation procedures, and evaluation of interobserver reproducibility in future prospective studies.

#### 4.2.4. Surgical Factors Influencing Long-Term Survival: Extent of Resection, Surgical Technique, and Postoperative Complications

The findings of our study, which included patients selected for surgical treatment, are consistent with recent evidence suggesting that survival differences between patients with solitary and multiple brain metastases may be less pronounced than previously believed. In carefully selected patients with multiple lesions, complete resection of all surgically accessible intracranial metastases, either during a single operative session or through a staged approach, may be associated with survival outcomes comparable to those observed in patients with a solitary brain metastasis [31].

Complete surgical resection has previously been associated with favorable outcomes in patients with brain metastases, including improved local control, overall survival, and preservation of neurological function. In our study, gross-total resection was associated with more favorable overall survival in the univariable analysis (*p* = 0.018). However, this association was not maintained after adjustment for other clinical and tumor-related variables in the multivariable Cox regression analysis. Specifically, subtotal resection was not associated with a significantly different hazard of death compared with gross-total resection (adjusted HR = 1.062, 95% CI 0.746–1.512; *p* = 0.737). Therefore, although the unadjusted findings are consistent with previous studies supporting maximal safe resection whenever feasible, extent of resection was not identified as an independent prognostic factor for overall survival in our cohort [1,5,31].

An important clinical advantage of surgical treatment in patients with symptomatic brain metastases is its ability to achieve immediate cerebral decompression, resulting in rapid relief of mass effect and associated intracranial hypertension. Furthermore, the integration of stereotactic radiosurgery into the perioperative treatment strategy, either in the preoperative or postoperative setting, may improve local tumor control, highlighting the complementary roles of surgery and radiosurgery in the contemporary management of brain metastases [26,27].

Patel et al. reported superior local control following en-bloc resection compared with piecemeal tumor removal [25,32]. In our cohort, however, surgical technique was not significantly associated with overall survival. Gross-total resection can be achieved using either an en bloc or a piecemeal approach, and the choice of technique often depends on tumor size, location, and accessibility, as well as its relationship with eloquent or critical neurovascular structures. Accordingly, our findings suggest that the specific resection technique may be more relevant to local disease control than to overall survival. Neither surgical technique nor extent of resection was independently associated with overall survival in our multivariable analysis.

The potential effect of extent of resection on local control was prospectively evaluated by Yoo et al. Using microscopic gross-total resection combined with the additional removal of a 5-mm margin of apparently normal peritumoral brain tissue, they reported a 1-year local recurrence rate of 29%, which was significantly lower than that observed following near-total resection (*p* < 0.01) [33]. These findings support the potential benefit of maximizing safe resection for local disease control but do not directly demonstrate an improvement in overall survival. Moreover, the available evidence remains heterogeneous. Stephanie T. et al. did not identify extent of resection as an independent predictor of either local recurrence or overall survival [34], highlighting the multifactorial nature of outcomes in patients with brain metastases.

Beyond its potential prognostic implications, surgical treatment remains particularly valuable in specific clinical scenarios. These include cystic metastases refractory to radiotherapy or systemic treatment and hemorrhagic metastatic lesions associated with acute neurological deterioration or life-threatening mass effect. In such situations, surgery provides rapid symptom relief and cerebral decompression and may represent the most effective therapeutic option for preventing further neurological decline and stabilizing the patient’s clinical condition [9,18].

## 5. Limitations

Our study has several limitations. First, its retrospective, observational, single-center design and the exclusive inclusion of patients selected for surgical treatment entail inherent risks of selection and information bias and preclude causal inference. In the absence of a non-surgical control group, the findings should be interpreted as prognostic associations identified within a surgically selected population rather than as evidence of a survival benefit attributable to surgery.

Second, the cohort was heterogeneous with respect to the origin and histology of the primary tumors, and detailed molecular characteristics were not consistently available. In addition, the study period from 2015 to 2023 encompassed substantial changes in systemic treatments, immunotherapy, targeted therapies, and perioperative radiotherapy strategies. Because many patients continued their oncological care at other institutions, detailed and standardized information regarding subsequent treatments could not be reliably obtained. Consequently, the inability to adjust fully for tumor biology, systemic disease activity, and subsequent oncological treatments represents an important source of residual confounding, particularly when interpreting long-term survival.

The extent of resection was determined primarily from the operative report and the surgeon’s intraoperative assessment rather than from contrast-enhanced MRI performed systematically during the early postoperative period.

Analyses incorporating postoperative variables also require cautious interpretation. KPS worsening was assessed on postoperative day 7, after the time origin of the survival analysis, which was defined as the date of surgery. Therefore, this variable may be influenced by surgical morbidity, postoperative complications, systemic deterioration, and pre-existing functional reserve and may introduce time-related bias. The 30-day mortality analysis was based on only 18 events; given the number of covariates evaluated, the logistic regression model may be susceptible to overfitting, unstable effect estimates, and limited precision.

Finally, patient-reported quality of life, neurocognitive performance, long-term functional recovery, local tumor control, intracranial recurrence, and progression-free survival were not systematically assessed. Prospective multicenter studies incorporating standardized imaging assessments, three-dimensional volumetric segmentation, molecular characterization, longitudinal documentation of oncological treatments, and patient-centered outcomes are required to validate these findings and improve their generalizability.

## 6. Conclusions

Brain metastases remain a complex clinical entity associated with considerable morbidity and significant therapeutic challenges in contemporary neuro-oncological practice. In our study, several clinical, imaging, and perioperative factors were associated with both early mortality and long-term survival following surgical treatment of brain metastases. A preoperative KPS score < 80, preoperative hydrocephalus, postoperative hematoma, and postoperative bronchopneumonia were associated with higher odds of 30-day mortality after adjustment for the other variables included in the model. Regarding overall survival, increasing age, a preoperative KPS score < 80, tumor volume ≥ 9 cm^3^, preoperative hydrocephalus, wound complications, and early postoperative KPS deterioration were significantly associated with shorter survival in the multivariable analysis.

Overall, our findings highlight the multifactorial nature of prognosis in patients undergoing surgery for brain metastases and emphasize the importance of careful patient selection, optimization of perioperative management, and prevention of postoperative complications. These results support a multidisciplinary therapeutic approach aimed at preserving functional status and improving both short- and long-term outcomes.

## Figures and Tables

**Table 1:** Patient demographics and clinical characteristics.

Characteristic	Category	N (%)
Number of patients		378
Age (years)	Median (range)	60 (18–83)
Gender	Male	221 (58.5)
Number of brain metastases	1	277 (73.3)
	2–3	68 (18.0)
	≥4	33 (8.7)
Tumor location	Supratentorial	269 (71.2)
	Infratentorial	63 (16.7)
	Supra- and infratentorial	46 (12.2)
Primary tumor	NSCLC	164 (43.4)
	Breast	55 (14.6)
	Melanoma	24 (6.3)
	CUP	67 (17.7)
	Kidney	16 (4.2)
	Colorectal	19 (5.0)
	Others	33 (8.7)
Extracerebral metastases	Present	103 (27.2)
KPS score	<80	138 (36.5)
	≥80	240 (63.5)
Time to diagnosis of brain metastases	Synchronous	150 (39.7)
	Metachronous	228 (60.3)
Comorbidities	Present	248 (65.6)
Hydrocephalus	Present	23 (6.1)
Clinical findings	Motor deficit	153 (40.5)
	Epileptic seizures	93 (24.6)
	Speech impairment	71 (18.7)
	Ataxia	83 (21.9)

**Table 2:** Multivariable binary logistic regression analysis of factors associated with 30-day mortality.

Variable	Adjusted OR	95% CI	*p*-Value
Preoperative KPS < 80	11.352	2.417–53.315	0.002
Hydrocephalus	7.226	1.093–47.772	0.040
Postoperative hematoma	24.416	6.121–97.400	<0.001
Postoperative pneumonia	27.944	5.623–138.872	<0.001

Abbreviations: OR—odds ratio; CI—confidence interval; KPS—Karnofsky Performance Score.

**Table 3:** Univariable Cox proportional hazards regression analysis of overall survival.

Variable	HR	95% CI	*p*-Value
Male sex	1.374	1.091–1.731	0.007
Age (per year)	1.016	1.004–1.028	0.009
Preoperative KPS < 80	1.340	1.070–1.690	0.012
Number of metastases	0.940	0.856–1.031	0.189
BM-CUP histology	0.930	0.690–1.240	0.607
Speech impairment	0.730	0.540–0.990	0.039
Tumor volume ≥ 9 cm^3^	1.380	1.050–1.820	0.023
Hydrocephalus	2.110	1.360–3.270	0.001
Subtotal vs. total tumor resection	1.430	1.060–1.930	0.018
Postoperative hematoma	2.690	1.620–4.450	<0.001
Postoperative CSF fistula	2.790	1.310–5.940	0.008
Postoperative KPS worsening	2.420	1.630–3.590	<0.001

**Table 4:** Multivariable Cox regression analysis of overall survival.

Variable	Adjusted HR	95% CI	*p*-Value
Age (per year)	1.020	1.007–1.034	0.003
Preoperative KPS < 80	1.645	1.262–2.144	<0.001
Tumor volume ≥ 9 cm^3^	1.618	1.210–2.162	0.001
Hydrocephalus	2.534	1.590–4.039	<0.001
Subtotal vs. total tumor resection	1.062	0.746–1.512	0.737
Wound complications	2.091	1.288–3.394	0.003
Postoperative KPS worsening	3.095	1.892–5.063	<0.001

Abbreviations: HR, hazard ratio; CI, confidence interval; KPS, Karnofsky Performance Status.

## Data Availability

The original contributions presented in this study are included in the article. Further inquiries can be directed to the corresponding author.

## Abbreviations

KPS	Karnofsky Performance Score
CUP	Cancer of unknown primary
OS	Overall survival
CVA	Cerebrovascular accident
CSF	Cerebrospinal fluid
BM-CUP	Brain metastases from cancer of unknown primary
WBRT	Whole-brain-radiotherapy
OR	Odds ratio
CI	Confidence interval
SRS	Stereotactic radiosurgery
